# Concentration of cadmium and lead in vegetables and fruits

**DOI:** 10.1038/s41598-021-91554-z

**Published:** 2021-06-07

**Authors:** Monika Rusin, Joanna Domagalska, Danuta Rogala, Mehdi Razzaghi, Iwona Szymala

**Affiliations:** 1 Department of Environmental Health, Faculty of Health Sciences in Bytom, Medical University of Silesia in Katowice, Piekarska 18, 41-902 Bytom, Poland; 2 Department of Mathematics, Bloomsburg University, Bloomsburg, PA 17815 USA

**Keywords:** Ecology, Plant sciences, Ecology, Environmental sciences, Natural hazards, Risk factors

## Abstract

Chemical contamination of foods pose a significant risk to consumers. A source of this risk is due to the consumption of products contaminated with heavy metals such as cadmium (Cd) and lead (Pb). The aim of the study was to research the levels of Cd and Pb contamination of selected species of vegetables and fruits in the form of fresh, frozen, dried and processed products. The goal was to verify which of these food groups was more contaminated with heavy metals. The study covered 370 samples of fruits and vegetables including apples, pears, grapes, raspberries, strawberries, cranberries, as well as beetroots, celeries, carrots and tomatoes. The content of Cd and Pb was determined by atomic absorption spectrometry. Quantitative results were analyzed using statistical models: analysis of variance, outlier analysis, post-hoc multiple comparison Tukey test. The tests showed that the levels of Cd and Pb concentration in samples of fresh, processed, frozen and dried fruits and vegetables varied substantially. The highest concentrations were recorded in dried products. Several fruit and vegetable samples exceeded the maximum permissible concentrations of Cd and Pb. The contamination of these products could be a significant source of consumer exposure to heavy metals when these products are a part of the diet.

## Introduction

Food quality and safety are perhaps the most important public health issues. Food available on the markets should be free of all chemical contaminants which pose a risk to consumer health, and its safety is not only the responsibility of food producers, but also state governments and agencies that systematically monitor and control food quality^1^. In Poland, this function is performed by the State Sanitary Inspection, which supervises food quality^2^.

A significant risk to the health of potential consumers is food contaminated with heavy metals, such as cadmium (Cd) and lead (Pb), exceeding the maximum permissible limits for food products. These limits are regulated by legal acts: Commission Regulation (EU) No 488/2014 of 12 May 2014 amending Regulation (EC) No 1881/2006 as regards maximum levels of cadmium in foodstuffs and Commission Regulation (EU) 2015/1005 of 25 June 2015 amending Regulation (EC) No 1881/2006 as regards maximum levels of lead in certain foodstuffs^3,4^. Heavy metals can be the cause of many chronic diseases whose symptoms are different depending on the level of toxicity of an element, as well as the duration and level of exposure^5,6^. Kidneys and liver are the main organs especially sensitive to Cd toxicity^7^. In the human body, Cd most often causes damage to both of these organs, as well as the testicles, lungs and bones. In addition, it causes a carcinogenic effect, initiating cancers of the prostate, kidneys, pancreas and testicles^5,8^. This element negatively affects the function of the skeletal system by disturbing the metabolism of calcium, magnesium, zinc, copper and iron ions^5,6^. In turn, Pb is a neurotoxic element. In the general public, but specially in children, elevated levels of Pb in the blood may cause changes in the brain, manifested by: lowering of the IQ level, a problem with proper perception and concentration and a hyperactivity^8,9^. Chronic exposure to Pb can be associated with an increased risk of developing neurodegenerative diseases^10^. Moreover, it has been demonstrated that Pb could have a role in the pathogenesis of deep vein thrombosis of lower limbs^11^.

In the case of vegetables and fruits, the source of their contamination with heavy metals may be the environmental conditions in which the cultivation was carried out^12^. The food product contamination with heavy metals may have also resulted from the migration of these elements from the packaging material. Contamination may have occurred during the technological processes that prepare the products for consumption, for example as the result of using metal kitchen tools^13,14^. In the case of foodstuffs stored in metal cans, the packaging coating may corrode, especially when stored foods have an acidic pH. Corroded metal packaging can become a source of migration of heavy metals, such as tin (Sn), Cd and Pb, to the stored product, that increase their content in food^15–17^. A human risk assessment can identify methods to minimize exposure to heavy metals, for example by reducing the weekly consumption of contaminated food products^18^.

According to the recommendations of the Food and Agriculture Organization of the United Nations, operating at the World Health Organization (FAO/WHO), the daily intake of fruits and vegetables for an adult should be at least 400 g − 5 times × 80 g per day^19^. Due to the seasonality of agricultural production, in countries located in the temperate climate zone there is a need to process food, freeze or dry it, especially those species of vegetables and fruits that are not available for sale all year. This procedure also applies to perishable food, such as soft fruits like strawberries or raspberries. Eating processed, frozen or dried fruits or vegetables can complement a daily diet, especially in periods, when the availability of fresh products is limited. However, not much scientific research concerns the quality of this type of food and its safety for the consumer.

The aim of this study is to investigate the level of heavy metals (Cd, Pb) contamination of particular species of vegetables and fruits, which are available on the Polish markets in the form of fresh, frozen, dried and processed products. These types of food could be a potential source of consumer exposure to heavy metals. In addition, the aim is to verify which of these food groups is the most contaminated and poses a significant health risk to its consumers.

## Material and methods

The study material consisted of 370 samples containing 6 species of fruits: apples (*Malus sylvestris* (L.) Mill. *var. domestica* (Borkh.) Mansf.), pears (*Pyrus communis* L.), grapes (*Vitis vinifera* L.), raspberries (*Rubus idaeus* L.), strawberries (*Fragaria* × *ananassa* Duchesne), cranberries (*Vaccinium macrocarpon* Aiton) and 4 species of vegetables: beetroots (*Beta vulgaris* L. *var. esculenta*), celeries (*Apium graveolens* L*. var. rapaceum*), carrots (*Daucus carota* L. *var. sativus*) and tomatoes (*Solanum lycopersicum* L.). Almost all species of fruits and vegetables were available on the Polish markets in the form of fresh, frozen, dried or processed. Processed fruits and vegetables were in form of 100% juice, compote, jam, marmalade or stored in syrup or marinade. The exceptions were frozen fruits which were not available for sale in Poland: apples, cranberries*,* grapes, and pears. The most frequent group was fresh food and in the following order: processed, dried and frozen (Table 1).

**Table 1:** The number of samples of particular types of vegetables and fruits.

Type of food	Sample	Number of samples				**Total**
		Fresh	Dried	Frozen	Processed	
Fruits	Apple	57	10	–	20	87
	Cranberry	4	5	–	4	13
	Grapes	15	11	–	4	30
	Pear	12	6	–	6	24
	Raspberry	7	4	4	4	19
	Strawberry	49	5	7	8	69
Vegetables	Beetroot	4	5	3	16	28
	Carrot	12	7	13	4	36
	Celery	21	4	5	9	39
	Tomato	10	5	4	6	25
	Total	191	62	36	81	370

The chemical analysis of heavy metals concentration (Cd and Pb) in 292 samples of vegetables and fruits was performed under the official food inspection carried out by the State Sanitary Inspection. The tests were performed by the Laboratory of Food Physicochemical Research operating at the Provincial Sanitary—Epidemiological Station in Katowice and by the Department of Laboratory at the District Sanitary-Epidemiological Station in Częstochowa. The tests were carried out as part of the official food control in accordance with the principles set out in chapter 3 of Regulation (EC) No 882/2004 of the European Parliament and of the Council of 29 April 2004 on official controls performed to ensure the verification of compliance with feed and food law, animal health and animal welfare rules^20^. The methods of sampling and analysis met the criteria specified of Commission Regulation (EC) No 333/2007 of 28 March 2007 laying down the methods of sampling and analysis for the official control of the levels of lead, cadmium, mercury, inorganic tin, 3-MCPD and benzo(a)pyrene in foodstuffs^21^. The remaining 78 samples were subjected to chemical analysis in the Analytical Laboratory operating at the Department of Environmental Health, Faculty of Health Sciences, Medical University of Silesia in Katowice. All mentioned laboratories are accredited in accordance with PN-EN ISO/IEC 17025:2005 by Polish Centre for Accreditation (PCA).

### Analytical methods

Fresh vegetables and fruits samples were prepared for chemical analysis in the same way that they are prepared for consumption (thorough washing, peeling, removing inedible parts). The cleared samples were shredded by a homogenizer T18 Digital Ultra-Turrax, IKA (Germany). The frozen samples were homogenized. The dried samples were grinded in a vibratory grinder Testchem PZS 01 (Poland). The processed products stored in syrup or marinade were separated and homogenized. The samples in the form of 100% juice, compote, jam and marmalade were thoroughly mixed. In the next phase, the samples were weighed using an analytical balance AS60/220/C/2, Radwag (Poland). The weight of each sample was varied and amounted to 2 g for fresh and frozen samples, 1 g for dried samples, 2 g for processed samples, except liquid samples—10 g. 8 mL of nitric acid (HNO_3_, 65%, Suprapur—Merck, Germany) was added to all food samples. Dried samples were further diluted by 1 mL 30% H_2_O_2_. Samples were subjected to a four-phases mineralization process in microwave mineralizer Magnum II, Ertec (Poland) (Table 2). Cd and Pb concentrations were determined by atomic absorption spectrometry (AAS)—SavantAA Sigma with PAL3000 automatic sample feeder and graphite furnace GF3000 (GBC, Australia). Measurements were made with atomization in graphite furnace and background correction; instrumental parameters and measuring range of the spectrometer AAS are summarized in Table 3. To estimate the limit of detection (LOD) and the limit of quantification (LOQ), at least 20 blank samples should be analyzed; subsequently mean value and standard deviation (SD) were calculated for these measures. LOD is the lowest quantity or concentration of a component that can be reliably detected with a given analytical method. It is estimated by:

$$ {\text{LOD}} = {\text{mean}}\,{\text{value}}\,{\text{of}}\,{\text{blank}}\,{\text{signals}} + \left( {3 \times {\text{SD}}} \right) $$

LOQ is the lowest analyte concentration that can be quantitatively detected with a stated accuracy and precision. Its estimate is given by:

$$ {\text{LOQ}} = {\text{mean}}\,{\text{value}}\,{\text{of}}\,{\text{blank}}\,{\text{signals}} + \left( {6 \times {\text{SD}}} \right) $$

LOD and LOQ values for Cd and Pb were summarized in Table 4. Standard solutions were used to create the calibration curve Certificate of Reference Material 1000 mg l^-1^ Lead Matrix: 2% HNO_3_ SPEX CertiPrep and Certificate of Reference Material 1000 mg l^-1^ Cadmium Matrix: 2% HNO_3_ SPEX CertiPrep standard solutions. Certified reference material (CRM)—Vegetable puree (TYG006RM) was used to confirm the correctness of analytical measurements in edible plant samples (Table 4).

**Table 2:** Microwave mineralization process.

Phases of mineralization process	Time (min)	Power (%)	Pressure (bar)	Temperature (°C)
I	10	60	17–20	295–300
II	5	80	30–32	295–300
III	10	100	42–45	295–300
IV*	10	–	–	–

*Cooling process.

**Table 3:** Instrumental parameters and measuring range for Cd and Pb in the spectrometer AAS.

Instrumental parameters/measuring range	Cd	Pb
Wavelength (nm)	228.8	217
Lamp current (mA)	5.0	0.3
Gap width (nm)	1.0	0.5
Measurement range (ppm)	0.0044–2.00	0.044–2.00
Values of the calibration curves (R^2^)	0.999	0.999

**Table 4:** Certified values of Certified Reference Materials (CRMs—TYG006RM) and LOD/ LOQ values.

Analyte	Reference value	LOD	LOQ
	(µg/l)		
Cadmium	289 ± 10	0.2	0.35
Lead	203 ± 10	1.7	3.5

### Statistical methods

Analysis of variance (ANOVA) was utilized to detect significant differences between average Cd and Pb concentrations in fresh, frozen, dried and processed vegetables and fruits. A p-value of less than 0.05 was used to indicate statistical significance. In the next stage, Outlier Analysis was performed. Boxplots were utilized to pictorially represent the location, dispersion and shape of the data distribution regarding the concentration of Cd and Pb in all types of analyzed food. Outliers show those values that largely differ from other values in the sample. They are typically either much higher or much lower than other points. Using the Post-hoc Multiple Comparison, attempts were made to determine significant differences between the mean values in the analyzed groups. Applying the Tukey’s HSD (Honestly Significant Difference) test, all pairs were compared (fresh-dried, frozen-dried, processed-dried, frozen-fresh, processed-fresh, processed-frozen) in four variants: Cd concentration in fruits, Cd concentration in vegetables, Pb concentration in fruits and Pb concentration in vegetables. The significance level was adjusted for multiple tests using Bonferroni correction^22^.

## Results and discussion

### Results of chemical analysis

The results of the study showed that the concentrations of Cd and Pb among all analyzed fruit samples (n = 242) were below the associated LOQs in only 87 and 96 samples, respectively. Similarly, in vegetable samples (n = 128) we found that Cd and Pb concentrations were below the LOQ in 31 and 69 samples, respectively. The levels of the Cd and Pb in the analyzed food samples were compared and contrasted with the maximum levels in foodstuffs regulated by legal acts: Commission Regulation (EU) No 488/2014 of 12 May 2014 amending Regulation (EC) No 1881/2006 as regards maximum levels of cadmium in foodstuffs and Commission Regulation (EU) 2015/1005 of 25 June 2015 amending Regulation (EC) No 1881/2006 as regards maximum levels of lead in certain foodstuffs^3,4^. It was found that in 12 food samples, the Cd content exceeded the maximum acceptable level. Among the fruit samples, this result was observed in: frozen raspberries (n = 1; 122% of maximum level) and frozen strawberries (n = 1; 114% of maximum level). In the case of vegetables, this result was observed in: fresh beetroots (n = 2; 203% and 670% of maximum level), frozen carrot (n = 1; 113% of maximum level), fresh celery (n = 4; 130%, 150%, 345%, 356% of maximum level) and processed tomatoes (n = 3; 102%, 112%, 134% of maximum level). The maximum permissible Pb level was exceeded in 3 analyzed food samples: fresh beetroot (n = 1; 135% of maximum level), frozen carrot (n = 1; 117% of maximum level) and 1 sample of frozen tomatoes in which the Pb concentration was up to 1074% of the acceptable limit (Table 5).

**Table 5:** The number and type of food samples in which the maximum level of Cd or Pb has been exceeded.

Type of food	Maximum level (mg/kg f.m.)		Type and number of samples with exceeded maximum level		% of maximum level	
	Cd^a^	Pb^b^	Cd	Pb	Cd	Pb
Raspberry	0.050	0.10	Frozen (1)	–	122	–
Strawberry	0.050	0.10	Fresh (1)	–	114	–
Beetroot	0.10	0.10	Fresh (2)	Fresh (1)	203 670	135
Carrot	0.10	0.10	Frozen (1)	Frozen (1)	113	117
Celery	0.20	0.10	Fresh (4)	–	130 150 345 356	–
Tomato	0.050	0.050	Processed (3)	Frozen (1)	102 112 134	1074

^a^According to Commission Regulation (EU) No 488/2014 of 12 May 2014 amending Regulation (EC) No 1881/2006 as regards maximum levels of cadmium in foodstuffs.

^b^According to Commission Regulation (EU) 2015/1005 of 25 June 2015 amending Regulation (EC) No 1881/2006 as regards maximum levels of lead in certain foodstuffs.

Tables 6 and 7 present the mean and SD, as well as the minimum and maximum values for the Cd and Pb contents in each of the analyzed fruits (Table 6) and vegetables (Table 7). Heavy metals concentrations were reported in mg/kg f.m. (fresh mass) in the fresh, frozen and processed products, while the content of Cd and Pb in dried products were presented in mg/kg d.w. (dry weight). Lack of a value in the tables means that the Cd or Pb value was below the LOQ for that particular sample.

**Table 6:** The mean value, standard deviation, minimum and maximum values ​​of Cd and Pb concentrations in particular types of fruit samples.

Samples	Mean (mg/kg f.m.)		SD		Min (mg/kg f.m.)		Max (mg/kg f.m.)	
	Cd	Pb	Cd	Pb	Cd	Pb	Cd	Pb
**Apple**								
Dried	0.023^a^	0.127^a^	0.0138^a^	0.0513^a^	0.009^a^	0.086^a^	0.039^a^	0.202^a^
Fresh	0.001	0.009	0.0017	0.0059	0.0004	0.001	0.0071	0.024
Processed	0.001	0.009	0.0002	0.0073	0.0004	0.002	0.001	0.025
**Cranberry**								
Dried	0.005^a^	0.009^a^	0.0039^a^	0.0028^a^	0.0016^a^	0.0071^a^	0.01^a^	0.011^a^
Fresh	0.008	0.004	0.0028	0.0012	0.005	0.003	0.011	0.005
Processed	0.006	0.01	0.0066	0.0007	0.001	0.009	0.015	0.01
**Grape**								
Dried	0.001^a^	0.047^a^	0.0005^a^	0.0349^a^	0.001^a^	0.0038^a^	0.0021^a^	0.1^a^
Fresh	0.001	0.005	0.0006	0.0031	0.0004	0.0006	0.002	0.009
Processed	0.0004	0.07	-	0.0805	0.0004	0.013	0.0004	0.127
Pear								
Dried	0.015^a^	0.036^a^	0.0008^a^	0.0035^a^	0.015^a^	0.033^a^	0.016^a^	0.038^a^
Fresh	0.004	0.008	0.0019	0.0052	0.001	0.008	0.0073	0.017
Processed	0.0008	0.0245	0.0004	0.0213	0.0005	0.004	0.001	0.047
**Raspberry**								
Dried	0.116^a^	0.111^a^	0.0056^a^	0.0266^a^	0.110^a^	0.092^a^	0.121^a^	0.129^a^
Fresh	0.011	0.012	0.0065	0.0115	0.003	0.003	0.021	0.033
Processed	0.009	0.011	0.0087	0.0049	0.002	0.007	0.019	0.014
Frozen	0.026	0.045	0.0232	0.0204	0.012	0.031	0.061	0.06
**Strawberry**								
Dried	0.131^a^	0.161^a^	0.1061^a^	0.0409^a^	0.037^a^	0.097^a^	0.277^a^	0.201^a^
Fresh	0.018	0.009	0.0152	0.0069	0.0009	0.002	0.057	0.027
Processed	0.003	0.006	0.0013	0.0034	0.0008	0.002	0.004	0.01
Frozen	0.016	0.011	0.0064	0.0015	0.006	0.009	0.024	0.012

^a^ [mg/kg d.w.].

**Table 7:** The mean value, standard deviation, minimum and maximum values of Cd and Pb concentrations in particular types of vegetable samples.

Samples	Mean (mg/kg f.m.)		SD		Min (mg/kg f.m.)		Max (mg/kg f.m.)	
	Cd	Pb	Cd	Pb	Cd	Pb	Cd	Pb
**Beetroot**								
Dried	0.048^a^	0.115^a^	0.0222^a^	0.2224^a^	0.021^a^	0.003^a^	0.072^a^	0.513^a^
Fresh	0.235	0.095	0.3003	0.0559	0.022	0.056	0.67	0.135
Processed	0.022	0.011	0.0147	0.0069	0.004	0.001	0.056	0.02
Frozen	0.027	0.173	0.0235	-	0.01	0.173	0.054	0.173
**Carrot**								
Dried	0.2^a^	0.206^a^	0.0997^a^	0.1319^a^	0.086^a^	0.09^a^	0.331^a^	0,348^a^
Fresh	0.041	0.027	0.0146	0.0121	0.024	0.02	0.062	0.041
Processed	0.034	0.018	0.0234	0.0152	0.004	0.004	0.054	0.034
Frozen	0.036	0.057	0.0320	0.0551	0.01	0.008	0.113	0.117
**Celery**								
Dried	-	0.259^a^	-	-	-	0.259^a^	-	0.259^a^
Fresh	0.152	0.031	0.2251	0.0267	0.0019	0.003	0.712	0.074
Processed	0.03	0.006	0.0241	0.006	0.012	0.0015	0.073	0.01
Frozen	0.044	0.064	0.0137	0.0223	0.026	0.048	0.062	0.08
**Tomato**								
Dried	0.103^a^	0.081^a^	0.1051^a^	0.0745^a^	0.032^a^	0.028^a^	0.285^a^	0.133^a^
Fresh	0.003	0.016	0.0028	0.0239	0.0006	0.0026	0.0058	0.052
Processed	0.038	0.031	0.0276	-	0.0059	0.031	0.067	0.031
Frozen	0.019	0.294	0.0038	0.3443	0.015	0.05	0.024	0.537

^a^(mg/kg d.w.).

The analysis of Cd and Pb contents in all food products is necessary due to the possibility of assessing the health risks associated with consumption of contaminated ready-to-eat different types of food. A review of the scientific literature showed that the issue of food contamination with heavy metals is discussed by several researchers. However, they mostly include only fresh fruits and vegetables. Additionally, there is a little data concerning the level of heavy metals contamination of vegetables and fruits cultivated in other European countries in the available literature. Consequently, the results presented in this paper may form the basis for further research on the scale of food contamination with heavy metals such as Pb and Cd.

Among fruits such as apples, pears, raspberries and strawberries, the highest average values of both Cd and Pb were observed in dried products (Cd: 0.023, 0.015, 0.116, 0.131 mg/kg d.w., respectively; Pb: 0.127, 0.036, 0.111, 0.161 mg/kg d.w., respectively). In cranberry samples, the highest levels of Cd were determined in fresh fruits (0.008 mg/kg f.m.), while Pb—in processed products (0.01 mg/kg f.m.). In the case of grape samples, the same average Cd concentration was recorded in both dried and fresh products (0.001 mg/kg), while the highest Pb content was observed in processed products (0.07 mg/kg f.m.). In most fruit samples the lowest average Cd concentrations were determined in processed products (grapes, pears, raspberries and strawberries—0.0004, 0.0008, 0.009, 0.003 mg/kg f.m., respectively), while Pb—in fresh fruits (cranberries, grapes, pears—0.004, 0.005, 0.008 mg/kg f.m.) or processed (raspberries and strawberries—0.011 and 0.006 mg/kg f.m.). In apple samples, the same average Pb value was recorded in both fresh fruit and processed products (0.009 mg/kg f.m.).

The content of Cd and Pb in fruits, in the results available in the literature, is very diverse. The demonstrated average Cd content in apples (0.001 mg/kg f.m.) is lower compared to studies from other regions of the world, including Great Britain (0.002 mg/kg f.m.)^23^. The amounts of Cd in raspberries and strawberries tested in Poland were higher compared to those investigated by Norton et al. (2015) (0.002 mg/kg f.m. vs 0.011 mg/kg f.m. and 0.002 mg/kg f.m. vs 0.018 mg/kg f.m.)^23^. Additionally, in samples collected in Turkey and Serbia, the Cd content in the analyzed products was below the LOQ^24,25^.

Our results of Pb values in fruit samples are similar to those reported by some researchers and the range of values presented for this element in other analyses were very wide. However, as in the case of Cd content in apples purchased in Poland, Pb concentrations in these fruits (0.009 mg/kg f.m.) were also lower than other studies—minimum of 200%^23^. The average Pb content in grapes (0.009 mg/kg f.m.) was comparable to that obtained by Bağdatlıoğlu et al. (2010) (0.006 mg/kg f.m.)^24^. The results of author's research regarding the content of Pb in raspberries (0.012 mg/kg f.m.) exceeded 2.5 times those published by Norton et al. (2015)^23^. Pb concentrations in strawberries (0.009 mg/kg f.m.) compared to other studies are in their lower range (0.010 mg/kg–0.027 mg/kg f.m.)^23,24^.

The highest average concentrations of Cd were determined in fresh vegetables (beetroot and celery—0.235 and 0.152 mg/kg f.m., respectively) and dried—carrots and tomatoes (0.2 and 0.103 mg/kg d.w.), while Pb—in frozen vegetables (beetroots and tomatoes—0.173 and 0.294 mg/kg f.m.), as well as dried (carrots and celery—0.206 and 0.259 mg/kg d.w.). For most samples, the lowest average Cd and Pb levels were observed in processed products (beetroots, carrots, celery). Exceptions were samples of tomatoes—the lowest average Cd and Pb concentration values were observed in fresh foodstuffs (0.003 and 0.016 mg/kg f.m., respectively).

Analyses conducted by other scientists indicate lower average Cd content in fresh beetroots (0.018–0.09 mg/kg f.m.)^23,26^ and higher by almost 600% in the case of Pb (0.58 mg/kg f.m.)^26^ compared to our research (Cd—0.235 mg/kg f.m.; Pb—0.095 mg/kg f.m.). Only the British study has shown lower Pb content (0.033 mg/kg f.m.)^23^. Our results—concentration of Cd (0.041 mg/kg f.m.) and Pb (0.027 mg/kg f.m.) in fresh carrot samples were similar to those obtained by other authors from the same territory in Poland, but also those from Great Britain, China or Brazil—Cd values ranged from 0.014 mg/kg f.m. to 0.03 mg/kg f.m., while Pb from 0.023 mg/kg f.m. to 0.971 mg/kg f.m.^23,26–28^. In the scientific literature we found only individual articles regarding celery heavy metal contamination. Guerra et al. (2012) showed 3 times lower Cd content in this vegetable—0.05 mg/kg f.m.^26^. The concentration of Pb in Brazilian research indicates higher content (0.47 mg/kg f.m.) than those obtained in this study (0.031 mg/kg f.m.)^26^. Tomatoes are the most frequently analyzed products, probably due to the easiness and simplicity of processing. Our analysis showed relatively low concentration of Cd and Pb in fresh tomatoes (Cd—0.003 mg/kg f.m.; Pb—0.016 mg/kg f.m.). In the most available scientific data Cd levels were in the range of 0.028 mg/kg f.m. to 0.033 mg/kg f.m., and Pb from 0.078 mg/kg f.m. to 0.18 mg/kg f.m.^26,28^. Only Norton et al. (2015) and Bagdatlioglu et al. (2010) noted lower or equal Cd and Pb values in the corresponding product^23,24^.

Massadeh et al. (2018) in Jordan determined Pb and Cd of various canned fruits and canned vegetables including canned juice (pineapple), canned tomato sauce, canned whole carrots and canned green beans. They showed metal concentration levels in the samples were in the range of 0.50–0.60 mg/kg f.m. for Cd and 2.6–3.0 mg/kg f.m. for Pb^29^. These results significantly exceed the values shown in present study, as well as the results presented by Domagała-Świątkiewicz and Gąstoł (2012) in the analysis of vegetable juices (beetroot, carrot, celery)^30^.

The high contamination found in vegetables might be closely related to the pollutants in irrigation water, farm soil, fertilizers and also industrial and low pollution household emissions. Differences in levels of contamination between fruits and vegetables may result from the specificity of the geographical area from which they are collected, their diverse capacity to accumulate heavy metals, as well as the way they are processed. It should be pointed out that in polluted environments (soil, water, and air), the presence of toxic metals in elevated concentrations is not uncommon. Due to the structure of consumption of various groups of food products both in Poland and other countries, a significant risk of exposure to heavy metals is associated with the consumption of fruits and vegetables, which are one of the main elements of the diet. Unfortunately, complete elimination of elements such as Cd or Pb from these products is impossible, and the technological processes used in food production can only remove a small part of the impurities from selected products or even contribute to their increased contamination. Thus, there is a need for regular monitoring of heavy metals on every kind of foodstuff, not only in fresh products, in order to estimate the health risk from heavy metals in the human food chain.

### Statistical analysis

#### ANOVA

For the purpose of ANOVA carried out to detect significant differences in the heavy metal concentrations of the four types of food (fresh, dried, frozen, and processed), samples with concentration value below the LOQ were removed from the analysis. In the case of Cd concentration, the value of F statistic was 11.15 for fruits and 4.049 for vegetables, leading to significant results with p-values below 0.001 and 0.01 respectively. For the of Pb concentration, the ANOVA results were even more extreme with F values of 56.59 for fruits and 7.13 for vegetables with associated p-values being below 0.001 in both cases. These results show that there is strong evidence to believe that mean Cd and Pb contents in the four types of fruits and vegetables are not equal (Table 8).

**Table 8:** Analysis of variance (ANOVA) for variates in four groups.

Variates	Type of food	Number of samples included in the analysis (excluded from analysis^a^)	Sum of squares	Mean of squares	F value*	Pr (> F) **	p value***
Cd concentration in fruits	fresh frozen processed dried	151 (86)	0.13887	0.00092	11.15	1.18e-06	< 0.001
Cd concentration in vegetables		90 (34)	1.415	0.01572	4.049	0.0095	< 0.01
Pb concentration in fruits		140 (93)	0.1030	0.00074	56.59	< 2e-16	< 0.001
Pb concentration in vegetables		55 (69)	0.5223	0.00950	7.138	0.000392	< 0.001

^a^Deleted due to value below the LOQ of the Cd or Pb concentration.

*The critical value for this test.

**The probability.

***The level of significance.

#### Outlier analysis

**Figure 1:**
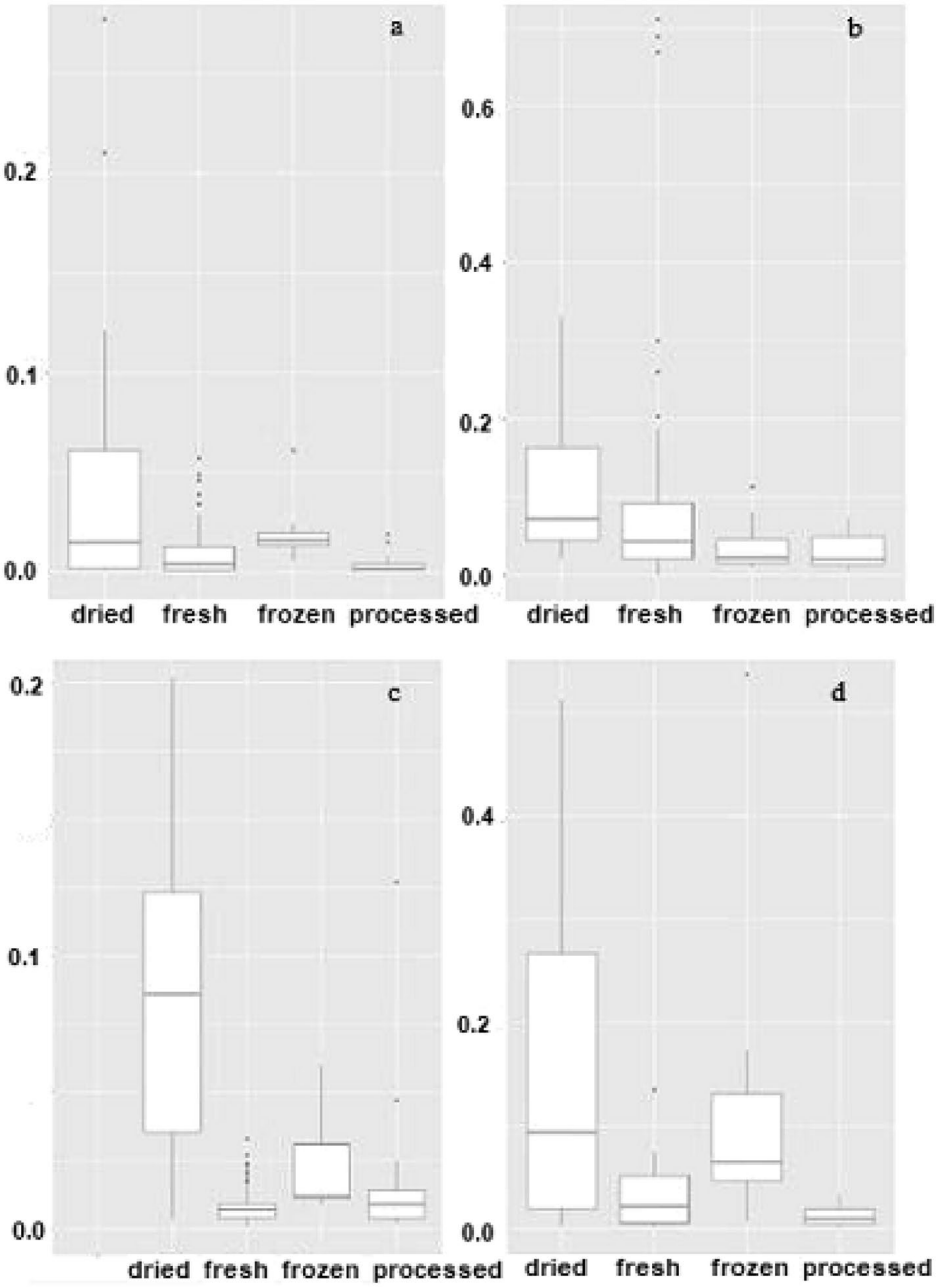
Outlier analysis in case: Cd concentration in: (**a**) fruits, (**b**) vegetables, and Pb concentration in: (**c**) fruits, (**d**) vegetables.

The boxplots depicted in Fig. 1 were used to illustrate the outlier analysis for Cd and Pb. Each plot shows the median of the observations along with the lower quartile (Q1) and the upper quartile (Q3). The highest and the lowest observations are shown by the whiskers. From Fig. 1a, there appears to be two outliers in the dried fruits with values 0.277 and 0.210. From Fig. 1b, there seems to be six outliers in the fresh vegetables with values of 0.203, 0.670, 0.260, 0.690, 0.300 and 0.712. In Fig. 1c, we see two outliers in the processed fruits with values of 0.127 and 0.047. Finally, Fig. 1d shows that there is one one outlier in the frozen vegetable category with the value of 0.537.

Outliers associated with high Cd and Pb values in fruit and vegetable samples may be the result of sample contamination during technological processes or vegetables/fruits cultivation in a polluted agricultural area.

### Post-hoc multiple comparison

**Figure 2:**
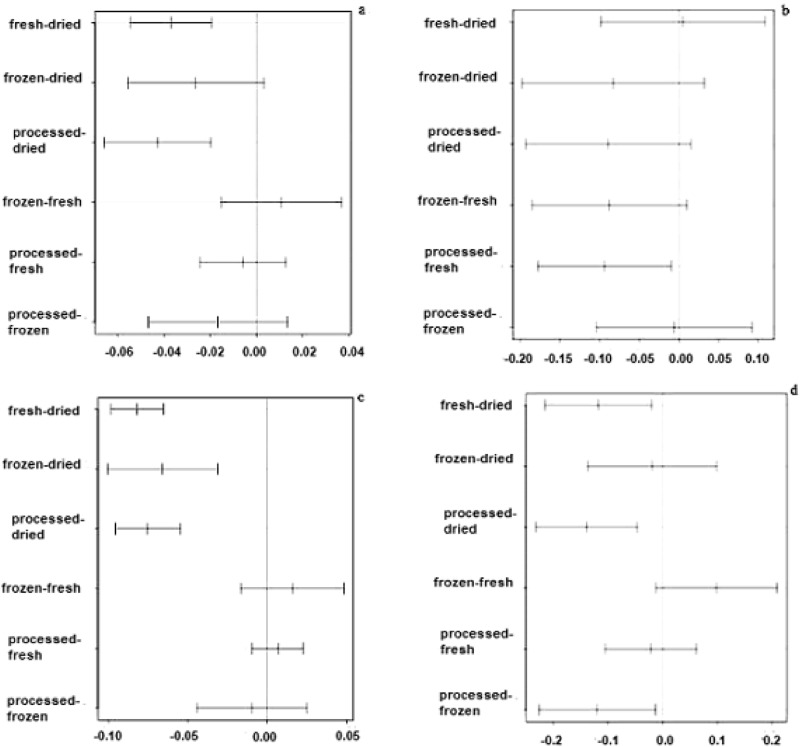
Post-hoc Multiple Comparison Tukey-Test of Cd and Pb in all samples of fruits and vegetables; differences in Cd mean concentration of: (**a**) fruits, (**b**) vegetables; differences in Pb mean concentration of: (**c**) fruits, (**d**) vegetables.

Since the ANOA results indicated significant differences among the mean concentrations of Cd and Pb both in fruits and vegetables, to further detect the specific different means, the Tukey HSD test^22^ was applied. The results are presented in Fig. 2. For the Cd concentration, comparison of all pairs of means indicated that the content of Cd in dried fruits is significantly different from mean concentrations of other types of food namely fresh, frozen, and processed fruits, see Fig. 2a. In the case of vegetables, the mean Cd contents of fresh and processed vegetables are different, see Fig. 2b, although mean Cd content of frozen and fresh vegetables are also significantly different if a significance level of 10% is used. Upon analyzing the mean concentrations of Pb in fruits, we found that the mean content of dried fruits was significantly different from the other three types, namely fresh, frozen and processed, see Fig. 2c. For the Pb concentrations in vegetables, a highly significant difference was detected between the means of processed and dried vegetables. In addition, mean Pb concentrations of fresh versus dried and processed versus frozen vegetables were significantly different, see Fig. 2d.

## Conclusions

The research showed that the values of Cd and Pb in all types of tested fruit and vegetable samples: fresh as well as processed, frozen and dried, are very diverse. The highest levels were noted in dried products. It may be the result of the elimination of water which increases the concentration of dry matter content. In addition, heavy metal contamination of dried fruits and vegetables may be the result of technological processes used in food production.

The maximum allowable concentration of the toxic metals was exceeded in several samples: Cd in 2 fruit and 10 vegetable samples, and Pb in 3 vegetable samples. Despite the fact that the exceedance of the Cd limit values concerned only 1% of the analyzed fruits and 9.3% of vegetables, and Pb—only 2.8% of all tested vegetables, the contamination of these groups of food products can be a significant source of consumer exposure to heavy metals, because they are an important part of the diet of most people.

The State Sanitary Inspectorate should thoroughly control those products in which the Cd and Pb limit values have been exceeded. It should be stated whether the exceedances occurred in only one batch of those products or if a larger number of them was contaminated. These products should be immediately withdrawn from sale and their producer should be obliged to indicate the source of their contamination.

## Data Availability

The datasets generated and analysed during the current study are available from the corresponding author on reasonable request.
